# Comprehensive analysis of recessive carrier status using exome and genome sequencing data in 1543 Southern Chinese

**DOI:** 10.1038/s41525-022-00287-z

**Published:** 2022-03-21

**Authors:** Jeffrey Fong Ting Chau, Mullin Ho Chung Yu, Martin Man Chun Chui, Cyrus Chun Wing Yeung, Aaron Wing Cheung Kwok, Xuehan Zhuang, Ryan Lee, Jasmine Lee Fong Fung, Mianne Lee, Christopher Chun Yu Mak, Nicole Ying Ting Ng, Claudia Ching Yan Chung, Marcus Chun Yin Chan, Mandy Ho Yin Tsang, Joshua Chun Ki Chan, Kelvin Yuen Kwong Chan, Anita Sik Yau Kan, Patrick Ho Yu Chung, Wanling Yang, So Lun Lee, Godfrey Chi Fung Chan, Paul Kwong Hang Tam, Yu Lung Lau, Kit San Yeung, Brian Hon Yin Chung, Clara Sze Man Tang

**Affiliations:** 1 Department of Paediatrics and Adolescent Medicine, LKS Faculty of Medicine, The University of Hong Kong, Hong Kong SAR, China; 2 Department of Surgery, LKS Faculty of Medicine, The University of Hong Kong, Hong Kong SAR, China; 3 Prenatal Diagnostic Laboratory, Department of Obstetrics and Gynaecology, Tsan Yuk Hospital, Hong Kong SAR, China; 4 Department of Paediatrics and Adolescent Medicine, Duchess of Kent Children’s Hospital, Hong Kong SAR, China; 5 Li Dak-Sum Research Centre, The University of Hong Kong–Karolinska Institute Collaboration in Regenerative Medicine, Hong Kong SAR, China

**Keywords:** Personalized medicine, Risk factors

## Abstract

Traditional carrier screening has been utilized for the detection of carriers of genetic disorders. Since a comprehensive assessment of the carrier frequencies of recessive conditions in the Southern Chinese population is not yet available, we performed a secondary analysis on the spectrum and carrier status for 315 genes causing autosomal recessive disorders in 1543 Southern Chinese individuals with next-generation sequencing data, 1116 with exome sequencing and 427 with genome sequencing data. Our data revealed that 1 in 2 people (47.8% of the population) was a carrier for one or more recessive conditions, and 1 in 12 individuals (8.30% of the population) was a carrier for treatable inherited conditions. In alignment with current American College of Obstetricians and Gynecologists (ACOG) pan-ethnic carrier recommendations, 1 in 26 individuals were identified as carriers of cystic fibrosis, thalassemia, and spinal muscular atrophy in the Southern Chinese population. When the >1% expanded carrier screening rate recommendation by ACOG was used, 11 diseases were found to meet the criteria in the Southern Chinese population. Approximately 1 in 3 individuals (35.5% of the population) were carriers of these 11 conditions. If the 1 in 200 carrier frequency threshold is used, and additional seven genes would meet the criteria, and 2 in 5 individuals (38.7% of the population) would be detected as a carrier. This study provides a comprehensive catalogue of the carrier spectrum and frequency in the Southern Chinese population and can serve as a reference for careful evaluation of the conditions to be included in expanded carrier screening for Southern Chinese people.

## Introduction

Carrier screening is a genetic test that allows prospective parents to determine the risk for recessive conditions in their offspring^1^. If both parents are carriers of the same autosomal recessive condition, there is a one in four chance they would conceive an affected child in each pregnancy^2^. As carriers are typically healthy and lack a family history suggestive of the disorder, most couples are unaware of their reproductive risks until they give birth to an affected child^3^. Prospective parents would benefit from carrier screening, to determine their risks of being carriers, their reproductive options, and to make informed decisions^3^.

Carrier testing as a screening test for single genetic conditions was first introduced in the 1970s for individuals with a family history of specific disease conditions or ethnicities with a higher prevalence of certain diseases^4^. Early implementation of screening included Tay-Sachs disease in the Ashkenazi Jewish population and β-thalassaemia in the Mediterranean population^4,5^. Carriers of Tay-Sachs disease and β-thalassaemia were screened via their reduced levels of hexosaminidase A enzyme and mean corpuscular volume respectively. The clinical utility of carrier screening was observed, Kaback et al. demonstrated a decrease in the incidence of Tay-Sachs disease after screening 2416 pregnant women with elevated risk^4^. Cost-benefit analysis of thalassaemia screening has also been previously reported in multiple studies demonstrating the economic benefit of preventing rare diseases^6–8^.

With the discovery of the *CFTR* gene in the 1980s, cystic fibrosis became one of the first conditions screened to undergo genetic screening^9,10^. Subsequent screening for autosomal recessive conditions was made possible via molecular testing methods. Organizations, such as the American College of Obstetricians and Gynecologists (ACOG) and the American College of Medical Genetics and Genomics (ACMG), then issued support to move from ethnicity-based screening to pan-ethnic screening for cystic fibrosis screening. Reasons for the change include limited access to carrier screening services for certain ethnicities, and complexities in assigning single ethnicities to individuals. Current ACOG and ACMG screening guidelines suggest pan-ethnic screening for spinal muscular atrophy and cystic fibrosis, and ACOG additionally suggests screening for haemoglobinopathies^11–13^.

The introduction of next-generation sequencing (NGS) has enabled effective simultaneous screening of multiple genes. With the use of NGS, gene-specific pan-ethnic carrier screening for recessive conditions can now be replaced by expanded carrier screening (ECS), which tests for hundreds of genetic conditions simultaneously at a reasonable cost. Multiple studies have identified carrier frequencies across various ethnicities^14–18^. The largest study carried out by Haque et al. examined carrier status in 94 disease conditions in 346,790 individuals of mixed ethnicities and demonstrated that ECS has increased the detection of carriers compared with racial- or ethnic- specific carrier screening according to recommendations from ACOG and ACMG^18^. The number of at-risk foetuses identified would be at least doubled compared with screening following professional guidelines, and in particular, 94 and 20% of fetuses at risk may be missed in East Asia and Southeast Asia, respectively^18–20^. This emphasizes the need for further investigation of ethnic-specific carrier data and the proportion of recessive conditions screened inside and outside of current professional guidelines.

While ECS can include as many genes as possible due to the advancement of NGS, ACOG and ACMG published a joint statement in 2015, emphasizing that proper selection of diseases to be included in the ECS panels is required^20^. It was suggested that the conditions included should have a well-defined phenotype with an early age of onset, which affects the quality of life detrimentally, results in cognitive/physical impairment, requires medical/surgical intervention, involves a prenatal diagnosis that results in interventions to improve perinatal/neonatal outcome/care, or necessitates prenatal education regarding special needs^20^. Two years later, ACOG suggested that conditions included in ECS should have, in addition to the criteria stated in 2015, a carrier frequency of at least 1 in 100. This aims to minimize anxiety associated with identifying rare conditions and the need for additional genetic testing and genetic counselling for families^21^. In 2021, ACMG released a practice resource outlining carrier screening guidelines for 97 autosomal recessive conditions using a tier system^22^. Currently, there is no consensus on which disease conditions should be included in ECS panels. The existing carrier screening research is primarily based on the Caucasian population or focused on the most prevalent conditions in different populations. Currently, information on the carrier frequency in Chinese is limited. While Zhao et al. performed ECS on 34 different Chinese ethnic groups; they evaluated carrier frequencies on 11 recessive conditions and determined a 27.49% carrier frequency which varied greatly between ethnic groups ranging from 4.15% to 81.35%^14^. Since a comprehensive assessment of the carrier frequencies in the Southern Chinese population is not yet available, we examined the spectrum and carrier status of 315 genes causing autosomal recessive disorders in 1543 Southern Chinese individuals; exome and genome sequencing data.

## Results

### Sequencing data characteristics

A total of 1543 unrelated, self-reported Southern Chinese individuals including 1116 with exome sequencing data (622 males and 494 females) and 427 with genome sequencing data (341 males and 86 females), passed sample-level QC procedures. This Southern Chinese cohort consisted of 963 males and 580 females and was used to evaluate the carrier status of recessive disorders in the Southern Chinese population. The Northern Chinese cohort, composed of 366 genome sequencing data with 294 males and 72 females, also passed sample-level QC procedures. This cohort was compared against the Southern Chinese cohort to reveal any Chinese subpopulation differences in carrier status.

The genes used for the evaluation of carrier frequency in this study were chosen by combining the gene list from 3 commercial companies as well as a gene list of treatable inherited diseases^23^. Among the 315 recessive genes evaluated in the sequencing cohort, 310 genes had at least 8 × mean coverage across the exonic region in >90% of the samples (Supplementary Fig. 1, Supplementary Data 1). The exceptions were *ADGRG1*, *MLC1*, *RMRP*, and *ELP1* in the exome sequencing data; and *CYP21A2* in both the exome and genome sequencing data. The total numbers of variants identified in the 315 recessive genes were 34,161 in exome sequencing data and 340,976 in genome sequencing data (Supplementary Table 1).

In this study, the carrier rate was estimated by combining the variants based on exome and genome sequencing data. Limited by the poor calling of copy number variations (CNVs) in the exome sequencing data, CNV calling was mainly performed on the genome sequencing data calibrated according to the CNV-JACG framework^24^. In addition to standard CNV calling algorithms, gene-specific bioinformatics tools were used for CNV calling of *SMN1*, *HBA1* and *HBA2* in the genome sequencing data^25–27^. Additional validation was performed for positive CNV calling cases of *SMN1* copy number and *HBA1*/*HBA2*.

### Carrier status of recessively inherited diseases in the Southern Chinese population

Among the 315 recessive genes, 353 variants (SNVs and small indels) and nine CNVs were classified as pathogenic or likely pathogenic (P/LP, Supplementary Data 2). In a total of 362 variants, 255 (70.4%) were loss-of-function mutations including frameshift, nonsense, splice-site, CNV, and start loss mutations. The remaining 107 (29.6%) variants included missense, in-frame, stop-loss and synonymous mutations (Supplementary Table 2). The most prevalent variant in our cohort was *GJB2* c.109G>A, which is associated with autosomal recessive deafness type 1A (Table 1). This variant was observed in 22.5% of the Southern Chinese population (*n* = 347), with 19 individuals carrying a homozygous variant for *GJB2* c.109G>A and three having a compound heterozygous mutation with another *GJB2* P/LP variant (Supplementary Table 3). Other common variants in descending order included the Southeast Asian deletion (–SEA) of the alpha thalassaemia genes (4.45%), the rightward deletion (-α3.7) of the alpha thalassaemia genes (3.04%), the *SMN1* exon 7 deletion (1.64%), and *GALC* c.1901T>C (1.43%). The top 15 most prevalent variants in the Southern Chinese population are listed in Table 2. In terms of disease condition, 11 diseases had a carrier rate of >1% in the Southern Chinese population (Table 1), and diseases that exceeded a 2% carrier rate included autosomal recessive deafness type 1A (carrier rate of 24.50%), followed by α-thalassaemia (carrier rate of 8.90%), spinal muscular atrophy type I (carrier rate of 2.11%), and systemic primary carnitine deficiency (carrier rate of 2.07%).

**Table 1:** Top 15 conditions with the highest carrier rates in the Southern Chinese population.

Condition	Gene	Count	Rate (%)
Deafness, Autosomal Recessive 1A; DFNB1A	*GJB2*	378	24.50
Thalassaemia, Alpha-	*HBA1/HBA2*	38	8.90^a^
Spinal Muscular Atrophy, Type I	*SMN1*	9	2.11^a^
Carnitine Deficiency, Systemic primary^b^	*SLC22A5*	32	2.07
Citrullinemia Type II, Neonatal-onset^b^	*SLC25A13*	28	1.81
Wilson’s Disease	*ATP7B*	27	1.75
Pendred Syndrome	*SLC26A4*	27	1.75
Krabbe Disease^b^	*GALC*	24	1.56
POLG-Related Disorders	*POLG*	20	1.30
Usher Syndrome, Type 2A	*USH2A*	18	1.17
Hb Beta Chain-Related Haemoglobinopathy (including Beta Thalassaemia and Sickle Cell Disease)	*HBB*	17	1.10
Glycogen Storage Disease, Type Ia	*G6PC*	14	0.91
Glycogen Storage Disease II	*GAA*	13	0.84
Phenylketonuria	*PAH*	12	0.78
Methylmalonic Aciduria due to Methylmalonyl-CoA Mutase Deficiency	*MMUT*	12	0.78
Cystic Fibrosis	*CFTR*	12	0.78

^a^Carrier rate was calculated from genome sequencing samples (*n* = 427) but not exome sequencing samples.

^b^Recessive Conditions not screened according to ACMG 2021 practice protocol.

**Table 2:** Top 15 allele frequencies of pathogenic variants identified in the Southern Chinese populations.

Gene	Condition	mRNA accession	Mutation Type	Nucleotide change	Protein change	dbsnp ID	Rate % (no. of samples)	Allele Frequency	gnomAD_EAS
*GJB2*	Deafness, Autosomal Recessive 1A; DFNB1A	NM_004004.6	Missense	c.109G>A	p.(Val37Ile)	rs72474224	22.49 (347)	0.11990	0.08345
*HBA1/HBA2*	Thalassaemia, Alpha-		–SEA				4.45 (19)^a^	0.02225^a^	
*HBA1/HBA2*	Thalassaemia, Alpha-		-α3.7				3.04 (13)^a^	0.01522^a^	
*SMN1*	Spinal Muscular Atrophy, Type I		*SMN1* exon 7 deletion				1.64 (7)^a^	0.0082^a^	
*GALC*	Krabbe Disease	NM_000153.4	Missense	c.1901T>C	p.(Leu634Ser)	rs138577661	1.43 (22)	0.00713	0.00830
*GJB2*	Deafness, Autosomal Recessive 1A; DFNB1A	NM_004004.6	Frameshift	c.235delC	p.(Leu79CysfsTer3)	rs80338943	1.36 (21)	0.00680	0.00652
*SLC25A13*	Citrullinemia Type II, Neonatal-onset	NM_001160210.1	Frameshift	c.852_855delTATG	p.(Met285ProfsTer2)	rs80338720	1.36 (21)	0.00680	0.00461
*SLC26A4*	Pendred Syndrome	NM_000441.2	Splice	c.919-2A>G	p.?	rs111033313	1.30 (20)	0.00648	0.00506
*POLG*	POLG-Related Disorders	NM_001126131.2	Missense	c.2890C>T	p.(Arg964Cys)	rs201477273	1.23 (19)	0.00616	0.00902
*HBB*	Hb Beta Chain-Related Haemoglobinopathy (including Beta Thalassaemia and Sickle Cell Disease)	NM_000518.5	Frameshift	c.126_129delCTTT	p.(Phe42LeufsTer19)	rs80356821	0.97 (15)	0.00486	0.00231
*SLC22A5*	Carnitine Deficiency, Systemic primary	NM_003060.4	Missense	c.1400C>G	p.(Ser467Cys)	rs60376624	0.91 (14)	0.00454	0.00226
*SLC25A20*	Carnitine-acylcarnitine translocase deficiency	NM_000387.6	Splice	c.199-10T>G	p.?	rs541208710	0.78 (12)	0.00389	0.00095
*SMN1*	Spinal Muscular Atrophy, Type I		*SMN1* silent mutation				0.47 (2)^a^	0.00234^a^	
*G6PC*	Glycogen Storage Disease, Type Ia	NM_000151.4	Synonymous	c.648G>T	p.(Leu216 = )	rs80356484	0.45 (7)	0.00227	0.00110
*PAH*	Phenylketonuria	NM_000277.3	Missense	c.721C>T	p.(Arg241Cys)	rs76687508	0.39 (6)	0.00194	0.00146
*ATP7B*	Wilson Disease	NM_000053.4	Missense	c.2975C>T	p.(Pro992Leu)	rs201038679	0.39 (6)	0.00194	0.00046
*SLC22A5*	Carnitine Deficiency, Systemic primary	NM_003060.4	Missense	c.51C>G	p.(Phe17Leu)	rs11568520	0.39 (6)	0.00194	0.00166
*SLC22A5*	Carnitine Deficiency, Systemic primary	NM_003060.4	Nonsense	c.760C>T	p.(Arg254Ter)	rs121908893	0.39 (6)	0.00194	0.00145

^a^Carrier rate was calculated from genome sequencing samples (*n* = 427) but not exome sequencing samples.

Southeast Asian deletion (--SEA) and rightward deletion (-α3.7) are the top two deletions responsible for α-thalassaemia.

Half of the 1543 Southern Chinese individuals (*n* = 737, 47.8%) were carriers of at least one recessive disorder (Table 3). It was found that 182 individuals (11.8% of the Southern Chinese population) were carriers for multiple diseases, with 152 of them (9.9%) being carriers of two recessive disorders. There were 21 individuals (1.4%) who were carriers of three recessive disorders, seven individuals (0.5%) for four recessive disorders, and two individuals (0.1%) who were carriers for five recessive disorders.

**Table 3:** Estimated burden of carriers in the Southern Chinese population.

Number of disease(s) carried in a person	Number of samples	Carrier rate (%)
0	806	52.2
1	555	36.0
2	152	9.9
3	21	1.4
4	7	0.5
5	2	0.1

The table shows the estimated burden of carriers in the Southern Chinese population. Approximately 47.8% (*n* = 737) of individuals were carrier of at least one recessive condition.

### Biallelic mutations present in the Southern Chinese population

*GJB2*-associated autosomal recessive deafness type 1A was the only condition in which biallelic mutations were identified in the same person (*n* = 22) in the study cohort. Among the 22 individuals with homozygous or compound heterozygous *GJB2* mutations, 15 had a clinical summary available, and five individuals were recorded to have mild to moderate hearing loss. Hearing loss was not a primary indication of recruitment for either exome sequencing or genome sequencing.

### Performance of commercial ECS panels in Southern Chinese

In this study, genes selected for evaluation were obtained from three commercial ECS panels and a list of genes that were considered treatable inherited conditions by Karnebeek et al.^23^. While the three commercial ECS panels have significant overlap among genes, some genes can only be identified in a specific ECS panel, ranging from three in the Myriad panel to 97 in the Invitae panel. When company-specific ECS panels were examined in the Southern Chinese population, the Invitae panel was able to detect 264 mutations in 693 carriers (94.0% of all carriers); the Baylor panel could detect 187 mutations in 643 carriers (87.2% of all carriers); and the Myriad panel was capable of detecting 200 variants in 635 carriers (86.2% of all carriers).

In Southern Chinese individuals, 1 in 12 individuals (8.30% of the population) were carriers for treatable inherited conditions according to Karnebeek et al., suggesting a high prevalence of carriers of treatable conditions in the Southern Chinese population. However, 32 genes that were associated with treatable inherited disorders were not included in any of the three commercial ECS panels, with a range of 32–53 genes absent in each ECS panel (Supplementary Fig. 2, Supplementary Data 1). When company-specific ECS panels were applied to our Southern Chinese cohort, 76 carriers (10.31% of all carriers), 69 carriers (9.36% of all carriers), and 15 carriers (2.04% of all carriers) for treatable conditions would have been missed by the Myriad panel, Baylor panel and Invitae panel, respectively.

### Founder mutations in Southern Chinese

Within this cohort, 3.1% (*n* = 11) of the identified mutations were previously known or suspected East Asian founder mutations (Supplementary Data 2). The eleven founder mutations were responsible for 13.3% (*n* = 98) of the identified carriers in this study. There were three founder variants with a carrier rate of larger than 1% being *HBA1*/*HBA2* –SEA deletion, *GJB2* c.235delC and *SLC26A4* c.919–2A>G with a carrier rate of 4.45%, 1.36% and 1.30% in the Southern Chinese population, respectively. The carrier rate for *HBA1*/*HBA2* –SEA deletion was determined using genome sequencing samples only. Other disease conditions with founder mutations identified in this cohort includes carnitine deficiency (*SLC22A5*), FKRP-related disorders (*FKRP*), glutaric acidemia IIC (*ETFDH*), Pompe disease (*GAA*), Hb Beta chain-related hemoglobinopathy (*HBB*), infantile neuroaxonal dystrophy 1 (*PLA2G6*), phenylketonuria (*PAH*), and Wilson disease (*ATP7B*).

### Northern and Southern Chinese carrier frequency comparison

In addition, using a small cohort consisting of 366 Northern Chinese individuals, we compared whether there were differences in the carrier frequency of certain recessive disorders. We calculated the 95% confidence intervals of the observed relative carrier frequencies per gene in both the Southern and Northern Chinese populations and used a two-sample z-test to compare the two proportions (Supplementary Data 3)^28^. Of the 55 genes with rare pathogenic variants found in both Southern and Northern Chinese individuals, *GJB2* (*p*-value: < 0.00001) was the only gene that had a significantly higher carrier frequency Southern Chinese individuals than in Northern Chinese individuals.

## Discussion

This is one of the first studies to comprehensively evaluate the carrier status in the Southern Chinese population with the secondary use of exome and genome sequencing data. In addition to the standard GATK pipeline, specific bioinformatics tools have been used to improve the deletion detection of *SMN1* and *HBA1*/*HBA2* in spinal muscular atrophy and thalassaemia, respectively. Our results demonstrated that 1 in 2 Southern Chinese individuals (47.8% of the population) was a carrier of at least one recessive disorder, with 0.1% of the population carrying up to five diseases conditions. In addition, 1 in 12 individuals (8.30% of the population) was a carrier of a treatable inherited condition, which was higher than that the rate among Singaporeans, at 1 in 18^29^.

The carrier rate of the most prevalent diseases in our cohort was compatible with published Southern Chinese data^14,30–32^. A direct side-by-side comparison across previously published carrier studies yields inconclusive results because of several reasons including different genomic technologies used, different reporting criteria, different disease conditions screened, and increased evidence of variant pathogenicity. A single disease condition comparison with existing literature such as the carrier rate of autosomal recessive deafness type 1A was found to be similar to that from Guangdong, which is a province of Southern China that shares population origin, culture, and language with Hong Kong^33^. The Southern Chinese carrier rate of pan-ethnic diseases such as spinal muscular atrophy was compatible with the 2% rate previously reported^34^. The carrier rate of alpha thalassaemia was found to be 8.90% in our study, which was higher than the previously reported rate of 5.0% in Hong Kong^35^. A possible reason for the discrepancy was that the carrier rate in our cohort was evaluated in genome sequencing data that had a small sample size (*n* = 427).

**Fig. 1:**
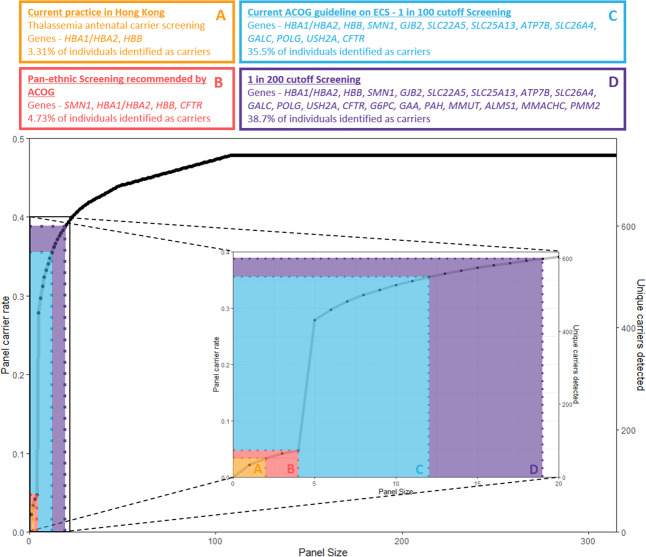
Incremental detection of using ECS panel. The figure shows the incremental benefits of using an ECS panel. The first tier of carrier screening includes α-thalassaemia and β-thalassaemia with 3.31% of individuals identified as carriers. This is based on Hong Kong’s current antenatal screening guidelines. The second tier includes the pan-ethnic carrier screening disease conditions recommended by ACOG which accumulates spinal muscular atrophy and cystic fibrosis. Using these criteria, 4.73% of individuals were identified as carriers. The third tier is based on the 2017 ACOG guidelines on ECS with a cut-off of 1 in 100. Using these criteria, 35.5% of individuals were identified as carriers. Mutations in *GJB2* were the most common, with a carrier rate of 24.5% in the Southern Chinese population (1 in 4 individuals). The fourth tier is a screening cut-off of 1 in 200, 38.7% of individuals were identified as carriers. Within this study, 47.8% of 1543 individuals were carriers of one or more recessive disease conditions.

By focusing on specific disease conditions based on currently available screening guidelines, we could observe the incremental benefit of using an ECS panel (Fig. 1). Currently, antenatal mean corpuscular volume thalassaemia screening is the only form of screening employed in Hong Kong^36^. Using 427 genome sequencing samples, we determined the carrier rates of thalassaemia to be 1 in 11 (8.9% of the population). Cystic fibrosis, spinal muscular atrophy and thalassemia are the only conditions selected for pan-ethnic carrier screening recommended by ACOG. The three disease conditions were in the top 15 conditions with the highest carrier rate in this cohort (Table 1). We determined the carrier rates of spinal muscular atrophy at 1 in 50 individuals (2.1% of the population) using genome sequencing samples and the carrier rate of cystic fibrosis was 1 in 129 individuals (0.78% of the population) in the Southern Chinese population. If the pan-ethnic carrier screening guidelines were used, 1 in 26 individuals (73 out of 1543, 4.73% of the population) would have been identified as carriers. If carrier testing is expanded according to the ACOG recommendation that conditions included for screening should have a carrier frequency of 1 in 100 or greater^21^, 11 disease conditions will meet the frequency threshold (Table 1), and 1 in 3 individuals (548 out of 1543, 35.5% of the population) was a carrier for these 11 conditions. If the 1 in 200 carrier frequency threshold is used, and additional seven genes would meet the criteria, and 2 in 5 individuals (598 out of 1543, 38.7% of the population) would be detected as a carrier. If carrier testing is further expanded to all 315 genes, 1 in 2 people (47.8% of the populations) will be a carrier for any recessive condition.

The incremental difference between screening guidelines shows the largest leap from pan-ethnic guidelines to the ACOG 1 in 100 carrier frequency (4.73% to 35.5%). This also suggests that when a threshold cut-off is not used, a significant proportion of carriers will be identified, which may bring anxiety associated with identifying rare conditions to the couple and require extensive counselling follow-up^21^. Therefore, further discussions are required to reach a consensus on the frequency threshold used in ECS to include conditions that are severe but relatively common in the local population. In the 97 autosomal recessive conditions suggested by the ACMG 2021 practice protocol for screening, a few conditions are more prevalent than 1 in 200 carrier frequency in Southern Chinese yet excluded from the list^22^. Examples include systemic primary carnitine deficiency, citrullinemia type II, Krabbe disease, and Alstrom syndrome, which are conditions that have a carrier frequency of at least 1 in 200 in the Southern Chinese population. Their carrier frequencies were 2.07%, 1.88%, 1.56%, and 0.65% respectively. Given their clinical severity and high carrier frequency, these conditions should be included in the recommendation for screening.

Based on the proposed framework of ACOG and ACMG^20^, there are a few variants in the Southern Chinese population that warrant further specification in carrier testing. Two of the most common variants identified in this cohort were a non-truncating *GJB2* variant c.109G>A and a truncating *GJB2* variant c.235delC. The two *GJB2* variants were found in 22.49% and 1.36% of Southern Chinese individuals respectively. These variants were classified as pathogenic by the ClinGen expert panel (Canonical Allele Identifier: CA172210; CA127025). All three commercial ECS panels included *GJB2* for testing; however, the variable expressivity of *GJB2* variants complicates the reporting of carrier status. Based on the different allelic combinations of truncating and non-truncating variants, varying levels of severity could be observed. It is known that 53% individuals carrying biallelic non-truncating variants develop mild hearing loss and 13% had profound hearing loss; 29–37% of individuals carrying biallelic truncating and non-truncating variants develop mild hearing loss and 24–30% have profound hearing loss; and 0–3% of individuals carrying biallelic truncating variants develop mild phenotypes and 59 to 64% develop profound hearing loss^37^. Because of a high *GJB2* carrier frequency, follow-up for non-syndromic hearing loss will be required in one-fourth of Southern Chinese individuals. Huang et al. determined that 36% of hearing loss patients with the c.109G>A homozygous variant or c109G>A plus another pathogenic variant developed severe to profound hearing loss^38^. While it could be argued that c.109G>A is a low penetrance, mild phenotype variant, a combination with a truncating variant such as c.235delC increases the likelihood of a more severe hearing loss^39^. Given that *GJB2* is typically screened in most carrier panels, this carrier information should be properly relayed back to the parents for them to have an informed understanding of their risks and possible future therapeutic procedures available. Therefore, genetic counselling should be included for parents to fully understand the risks of the *GJB2* variants given the complex variable expressivity and penetrance. Additionally, ECS should have an opt-out option for parents in screening mild phenotypes, variable expression, and low penetrance variants to prevent unnecessary anxiety as stated by the 2013 ACMG statement^40^.

In this cohort, we identified a carrier rate of 1.56% in Krabbe disease. The majority of patients with Krabbe disease typically have disease onset before 1 year of age^41^, which fulfils the ACOG and ACMG criterion that included diseases should have early onset in life^20^. However, the *GALC* c.1901T>C identified, which had a carrier frequency of 1.43% and was the fifth most common variant in the Southern Chinese population, was known to be associated with late-onset Krabbe disease with mild phenotypes^42,43^. Late-onset Krabbe disease ranges from late-infantile (7 months to 3 years), juvenile (3–8 years) to adult (≥ 9 years). Bascou et al. summarized a list of previously reported in literature *GALC* c.1901T>C variants in combination with other *GALC* pathogenic variants^44^. The age of onset in cases with *GALC* c.1901T>C as one of the alleles ranged from infantile onset at 7 months to adult onset at 40 years old. The carrier rate in our study matches our current understanding of the variant allelic frequency in gnomAD East Asians with a rate of 0.0083 and a carrier frequency of 1.66%. Using Hardy-Weinberg equilibrium, an estimation of Krabbe disease prevalence based on the allelic frequency of *GALC* c.1901T>C in Southern Chinese would be approximately one in 20,000. This is an increased disease prevalence of Krabbe disease compared to the currently reported rate of one in 250,000 in the United States and one in 100,000 in Europe^45,46^. This could be explained by the late onset and less severe nature of the c.1901T>C variant and that it has only been previously reported in Asian countries^47^. Adult physicians should pay more attention to the possibility of late-onset Krabbe disease due to the elevated allelic frequency in the Southern Chinese population. Despite the variable onset nature of Krabbe disease, it should still be included in the ACMG 2021 practice protocol due to the high carrier frequency with previous reports of infantile and juvenile age of onset. Further recommendations by professional bodies on how to report variants known to be associated with late-onset disease phenotype and the pre-test and post-test genetic counseling will be required.

ECS is now easily accessible and offered by many commercial laboratories. We compared the three commercially available ECS panels with a list of conditions that were defined as treatable inherited conditions by Karnebeek et al.^23^. We found that different ECS panels included different lists of genes, and remarkably, 32 genes associated with treatable disorders were not included in all three commercial ECS panels, suggesting that there was no consensus regarding which conditions to be include in carrier screening. When applying different ECS panels to the Southern Chinese population, the number of carriers identified varies. Therefore, the choice of ECS panel may affect the report of carrier status of the tested participants; that is, a participant may not be a carrier for recessive disease in one ECS panel but could be a carrier when another ECS panel is used.

A recent study by Cheng et al. investigated the current perspective on ECS in Hong Kong and determined that 70.7% of non-pregnant women accepted ECS hypothetically compared to 61.2% of pregnant women^48^. The findings found in Hong Kong was consistent with previously published international studies. The study also found 94% of women perceived ECS as least as effective or superior compared to traditional screening methods. Furthermore, Johansen Taber et al. described the clinical utility of ECS and the impact on reproductive outcomes of at-risk couples defined as both partners carry pathogenic variants in the same gene^49^. Changes in reproductive actions were seen in both at-risk couples screened during preconception period and prenatal period. Further investigations will be required before the implementation of ECS in a clinical setting.

With the advancement of bioinformatics tools, it is now possible to detect CNVs from NGS data, or even distinguish a targeted gene from genes with high sequence homology. First, NGS4THAL was used to detect the deletions of *HBA1 and HBA2*^27^. As a result, in addition to the four carriers who had SNVs that were detected by the standard bioinformatics pipeline, 34 carriers with *HBA1*/*HBA2* deletions were identified. Second, *SMN1* deletions were detected by SMAca and SMNCopyNumberCaller^25,26^. Because of the high sequence similarity between *SMN1* and *SMN2* and the complexity of the SMN locus, the detection of *SMN1* CNVs from NGS data is difficult. SMAca and SMNCopyNumberCaller are tools optimized to overcome the technical difficulties in detecting *SMN1* deletion from NGS, and the use of these tools allowed the detection of 33 carriers who had *SMN1* deletions, which were all missed by the standard GATK workflow. Furthermore, SMAca and SMNCopyNumberCaller were able to call the *SMN1* silent carrier through utilizing the detection of the highly correlated *SMN1* c.*3+80T>G variant. In the future, CNVs and structural variations could be more reliably identified from exome data with updated bioinformatics tools.

The study cohort was comprised of individuals enrolled for rare disease or complex disease research in exome sequencing and Hirschsprung’s disease in genome sequencing samples. To reduce potential biases of the recruitment cohort, the study design removed individuals with pathogenic variants as carriers in recessive condition congruent with their primary indications. Due to the nature of conducting secondary findings on next-generation sequencing samples with primary indications, the carrier frequencies identified in this study will only provide an estimate of the genuine carrier frequency of the Southern Chinese population. Further studies on a large healthy control population cohort will be required to estimate the genuine carrier frequency.

In this study, CNVs were evaluated in the genome sequencing data but not the exome sequencing data since the detection of CNVs in exome data is inconsistent among different methods^50^. Carrier rates of X-linked conditions were also not studied due to high proportion of males in the cohort. In addition, with the advancement of bioinformatics, we were able to detect large deletions in *SMN1*, *HBA1*, and *HBA2* in the genome sequencing data, which was not possible using standard CNV calling algorithms. The carrier rate of spinal muscular atrophy detected in this study, a pan-ethnic disease, was compatible with the 2% previously reported^34^. However, there were limitations in validating the silent carrier mutations of *SMN1* using conventional MLPA methods. The carrier rate of alpha thalassaemia was found to be 8.90% in this study, which was higher than the previously reported rate of 5.0% in Hong Kong^35^. Since the sample size of the genome sequencing data is small (*n* = 427), the carrier rates of thalassaemia and spinal muscular atrophy should be interpreted with caution. In the future, the advancement of exome CNV calling algorithms may facilitate the detection of CNVs and structural variations from exome data.

Third, we compared whether there were differences in the carrier frequency of certain recessive disorders between Southern and Northern Chinese individuals and found that *GJB2* was the only gene found to have significant difference (Supplementary Data 3). Since the sample size of the Northern Chinese population was small (*n* = 366) compared to that of the Southern Chinese population (*n* = 1543), we may not have enough statistical power to detect other genes with significant differences. For example, thalassaemia has a much higher prevalence in Southern Chinese individuals than in Northern Chinese individuals due to the presence of the –SEA founder mutation^51^, whereas phenylketonuria is more common in Northern Chinese individuals^52^. Therefore, it is important to carry out population-based studies in Northern Chinese individuals to evaluate their carrier spectrum.

With the use of exome and genome sequencing data from 1543 Southern Chinese individuals, the carrier status for 315 recessive genes was evaluated. It was found that 1 in 2 people (47.8% of the population) was a carrier for any recessive condition, and 1 in 12 individuals (8.30% of the population) was a carrier for treatable inherited conditions. Our results could serve as a reference to evaluate the conditions to be included in expanded carrier screening in Southern Chinese individuals.

## Methods

### Subjects and sequencing

A total of 2095 unrelated, self-reported Chinese individuals were enrolled for rare disease diagnosis or complex disease research from 2012 to 2019. Exome sequencing was performed in 1141 individuals, whereas genome sequencing was performed in 956 of them. Sequencing was performed on genomic DNA derived from peripheral blood or buccal mucosa by Illumina sequencing platforms. The details of the exome sequencing cohort have been previously described and used to evaluate the spectrum and prevalence of incidental findings and pharmacogenetics in the Southern Chinese population^53,54^. The genome sequencing cohort consisted of 956 Asian individuals and was used to study the genetics of Hirschsprung’s disease in Asia^55^. If an individual was identified as a carrier for a recessive condition congruent with the primary indications for referral, they were not counted as carrier in this study.

The paired-end sequencing reads were processed by a pipeline based on the Genome Analysis Toolkit (GATK) version 3.4^56^. Briefly, reads were aligned to the University of California Santa Cruz (UCSC) hg19 reference genome assembly by the Burrows-Wheeler Aligner, and duplicated reads were removed by Picard^57,58^. Local realignment around indels, base quality score recalibration, and cohort-based multisample variant calling were performed using the GATK toolset. Both exome and genome sequencing datasets were subjected to stringent quality control (QC) procedures. At the sample level, a sample check was performed using PLINK 1.90 beta 5.3 or Peddy and duplicated or first-degree related samples were removed from further analysis^59,60^. Principal component analysis was performed using Peddy to compare with 1000 Genomes Project reference data to remove samples not clustered with the East Asian population^61,62^. As a result, 1116 Southern Chinese exome sequencing data samples passed sample QC, whereas for genome sequencing, 427 Southern Chinese and 366 Northern Chinese samples remained for further analysis. Following variant level QC, variants with genotype quality <20 and read depth <8× were removed by KGGseq^63^, and variants also had to pass Variant Quality Score Recalibration (VQSR) annotated by GATK with a SNP tranche sensitivity threshold of 99.5% and INDEL tranche sensitivity threshold of 99.0%. The variants were annotated using ANNOVAR (Build 20200223)^64^ with the dbNSFPv4.0a database.

For genome sequencing data, variant calling of CNVs was performed with four complementary tools, which included CNVnator, Delly, Lumpy, and Seeksv^65–68^. This was calibrated according to the CNV-JACG framework with CNVs passing all filters with the “PASS” mark^24^. Because of the high sequence homology of pseudogenes and locus complexity, gene-specific bioinformatic tools were used for CNV calling for *SMN1* and *HBA1*/*HBA2*, which are the disease-causing genes of spinal muscular atrophy and alpha thalassaemia, respectively. CNV calling for *SMN1* was performed on genome sequencing data by both SMAca and SMNCopyNumberCaller which utilizes differentiating bases between *SMN1* and *SMN2*^25,26^; whereas CNV calling for *HBA1* and *HBA2* was performed by NGS4THAL on genome sequencing data^69^.

### Selection of recessive genes for evaluation of carrier status

To examine the spectrum of pathogenic variants in the Chinese population, genes included in three commercially available expanded carrier screening panels were selected. In addition, 104 genes that were associated with treatable inherited diseases, which were defined by Karnebeek et al., were also included^23,29^. This resulted in a list of 315 genes (Supplementary Data 1), where genes related to mitochondrial inheritance, X-linked conditions and autosomal dominant diseases were excluded from our analysis.

### Variant and CNV classification

Manual review and classification of the variants identified in the 315 genes were performed regardless of the pathogenicity stated in the ClinVar (version 20190305) and Human Gene Mutation Database (HGMD) (version 201701). Variants in the noncoding regions were excluded from analysis unless they have previously been reported as pathogenic/likely pathogenic in ClinVar or DM/DM? on HGMD. In-silico prediction tools used for the computational evidence of deleterious effects include CADD^70^, REVEL^71^, SIFT^72^, PolyPhen2 HDIV^73^, MutationAssessor^74^, FATHMM^75^, PROVEAN^76^, and LOFTEE^77^. Variants (single nucleotide variants and small indels) were classified according to the ACMG and the Association for Molecular Pathology (AMP) guidelines for sequence variant interpretation as the framework^78^. Disease-specific ClinGen variant interpretation guidelines were used for variant classification in hearing loss, phenylketonuria, and lysosomal storage disorders^79–82^. In addition, loss of function variants were classified according to the ClinGen recommendations for interpreting the loss of function PVS1 criterion^79,83^. Finally, CNVs were classified according to the ACMG and ClinGen guidelines for constitutional CNVs^84^.

### *SMN1* validation

Validation of *SMN1* deletions was performed using the SALSA^®^ MLPA^®^ probemix P060 SMA Carrier (MRC Holland) according to the manufacturer’s protocol. The results of the validation are described in the supplementary information.

### *HBA1/HBA2* validation

While –SEA variants were called consistently by both NGS4THAL and CNV-JACG, validation was performed only on -α3.7 and -α4.2 deletions using the SALSA^®^ MLPA^®^ probemix P140 HBA (MRC Holland) according to the manufacturer’s protocol. The results of the validation are described in the supplementary information.

### Reporting summary

Further information on research design is available in the Nature Research Reporting Summary linked to this article.

## Supplementary information

Supplementary Information

Gene list and coverage of the coding regions of the 315 recessive genes

List of pathogenic variants identified in the Southern Chinese populations

Comparison of genes with differences in carrier frequency in the Southern and Northern Chinese populations

Reporting summary

## Data Availability

The datasets for this article are not publicly available due to concerns regarding participant/patient anonymity. Requests to access the datasets should be directed to the corresponding author. Data available from the corresponding author will be de-identified before the data is handed over to qualified researchers.

## Code availability

Processing of raw sequence files, variant calling, variant annotation, and all other analyses described in this study were performed using available and open source software, as described in the “Methods” section. No custom code was therefore used, apart from simple scripts integrating commands for such programs.
