# Supplementary material for: Comprehensive analysis of recessive carrier status using exome and genome sequencing data in 1543 Southern Chinese
Source: NPJ Genom Med. 2022 Mar 21;7:23. doi: 10.1038/s41525-022-00287-z (PMC8938515; doi:10.1038/s41525-022-00287-z)
Supplement: Supplementary file 1 — Supplementary Information [file 41525_2022_287_MOESM1_ESM.pdf]

Supplementary Information

SMN1 validation

In this study, copy number variations (CNVs) calling for *SMN1* was performed on genome sequencing data by both SMAca and SMNCopyNumberCaller [1, 2]. Eleven carriers were detected by the two tools, among which nine of them could be detected by both tools, including two silent carriers. One sample was detected by SMAca but not SMNCopyNumberCaller; whereas one sample was detected by SMNCopyNumberCaller but not SMAca.

Multiplex ligation-dependent probe amplification (MLPA) validation has been performed (n=9) except for the two silent carriers, because MLPA cannot detect *SMN1* silent mutation. MLPA was able to validate and confirm the seven carriers which were detected by both the SMAca and SMNCopyNumberCaller. For the remaining two cases which were detected by either one of the tools, they could not be validated by MLPA and hence were considered false positive. Since the two silent carriers were detected by both tools, we considered it as likely true positive. The number of carriers for spinal muscular atrophy in the Southern Chinese was nine, and the carrier rate was 2.11%.

The copy number of *SMN1* and *SMN2* were determined using SMA MLPA [SALSA MLPA Probemix P060-B2 SMA Carrier, (MRC-Holland)]. The SMA MLPA assay was performed according to the manufacturer's intended use instructions. The assay results were analyzed using Coffalyser.Net software v5.3 to determine the copy number of the *SMN1* and *SMN2*.

HBA1/*HBA2* validation

In this study, detection of *HBA1*/*HBA2* deletions was performed on genome sequencing data by NGS4THAL. In total 34 carriers were detected by the bioinformatic tool, with 19 individuals carrying the –SEA mutation, 13 individuals carrying the a3.7 mutation, and 2 individuals carrying the a4.2 mutation. The copy number of *HBA1*/*HBA2* was determined using MLPA [SALSA MLPA Probemix P140 HBA, (MRC-Holland)].

Supplementary References:

1. Lopez-Lopez, D., et al., *SMN1 copy-number and sequence variant analysis from next-generation sequencing data.* Hum Mutat, 2020. **41**(12): p. 2073-2077.
2. Chen, X., et al., *Spinal muscular atrophy diagnosis and carrier screening from genome sequencing data.* Genet Med, 2020. **22**(5): p. 945-953.

Supplementary figures

Supplementary Figure 1 – Coverage plot of ES and GS under 8×, 20× and 30×

(A) Coverage of ES on 315 genes

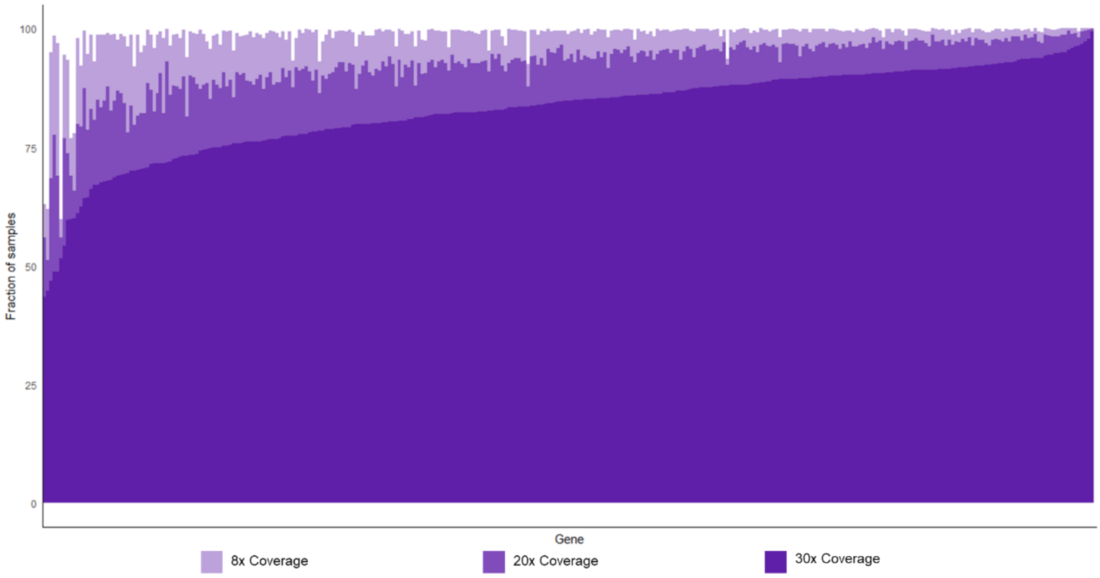

The plot shows the fraction of samples with at least 8×, 20×, and 30× mean coverage in the exonic region of the 315 recessive genes in the exome sequencing data. Over 90% of samples attained a minimum of 8× coverage across the exonic region for all genes except the *ADGRG1* (59.9%), *MLC1* (61.9%), *RMRP* (63.0%), *ELP1* (76.9) and *CYP21A2* (77.9%) genes.

(B) Coverage of GS on 315 genes

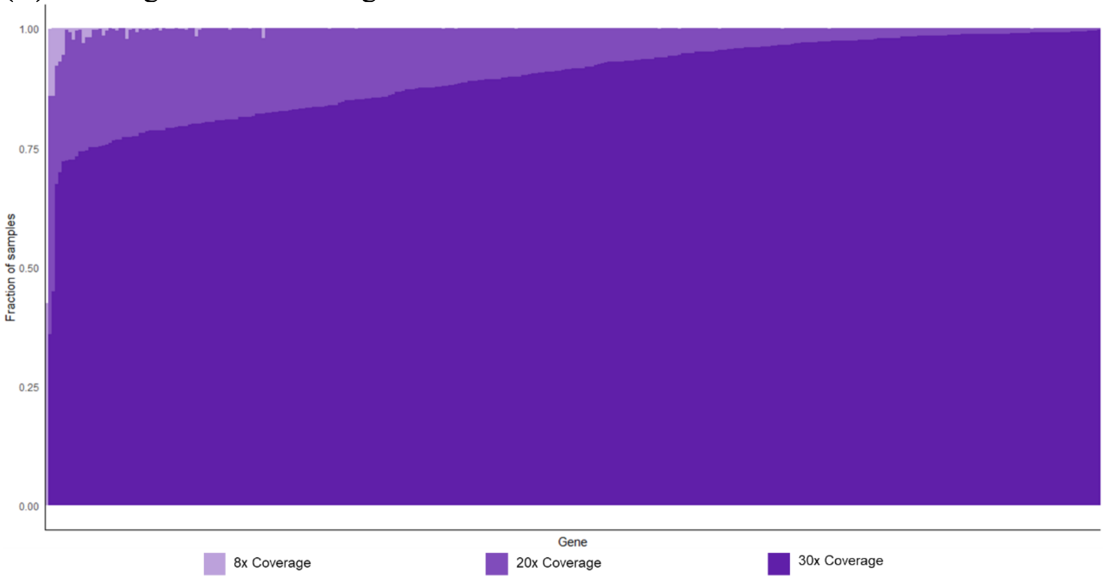

The plot shows the fraction of samples with at least 8×, 20×, and 30× mean coverage in the whole gene for the 315 recessive genes in the genome sequencing data. Nearly all samples attained a minimum of 8× coverage across the whole genomic region for all genes except the *CYP21A2* gene (42.4%).

Supplementary Figure 2 – Venn diagram of the three commercial panels (Baylor, Myriad, and Invitae) and medically actionable genes included in one research study (Karnebeek et al.)

(A) Gene set

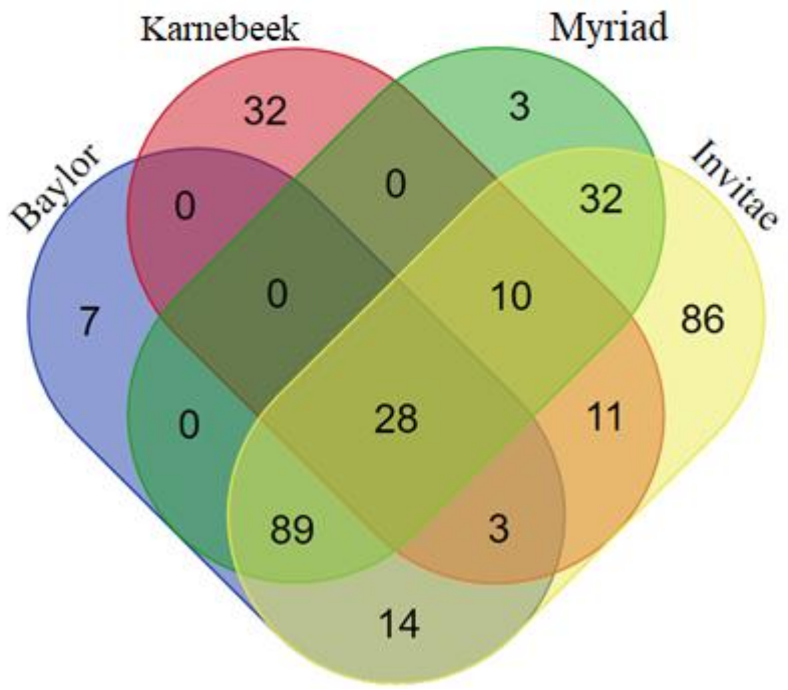

(B) Number of carriers identified

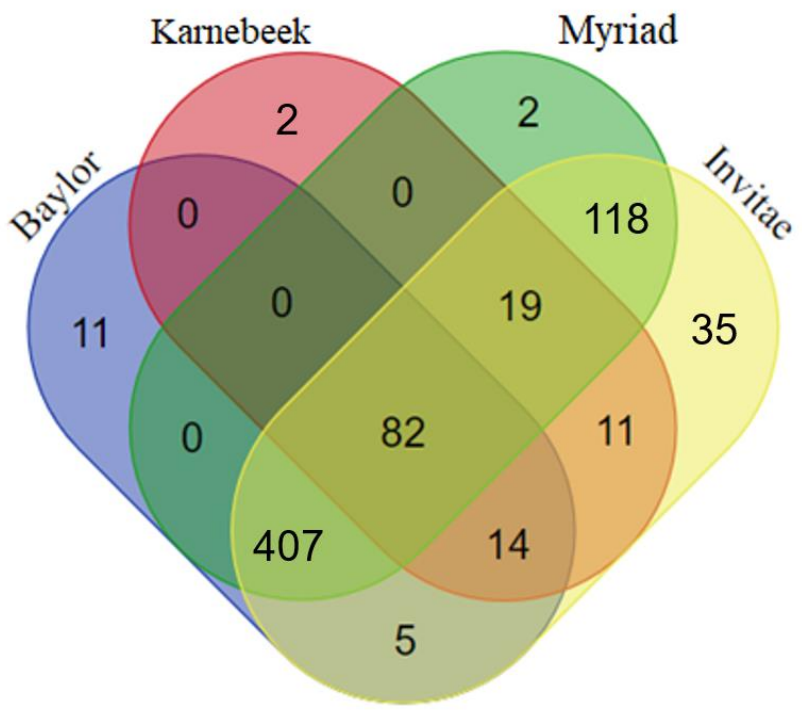

Supplementary Tables

Supplementary Table 1: The spectrum of variants identified in the exome and genome sequencing data

The table shows the number of variants identified in exome and genome sequencing separated by their predicted function.

**Function**	**Exome sequencing (n=1,116)**	**Genome sequencing (n=793)**
UTRs (5’ and 3’)	2,349	7,431
Intronic	24,201	317,713
Coding	3,808	9,615
Protein-altering	3,760	6,142
Splicing	43	75
Total	34,161	340,976

Supplementary Table 2 - Spectrum of pathogenic variants identified in the Southern Chinese population

This table shows a summary of the pathogenic carrier variants identified in 1543 Southern Chinese cohort sorted by variant function type.

**Variant type**	**Number of variants (%)**
Frameshift	127 (35.1)
Missense	99 (27.3)
Nonsense	72 (19.9)
Splice	45 (12.4)
CNV	9 (2.49)
In-frame (deletion)	4 (1.10)
Start loss	2 (0.55)
Intronic	2 (0.55)
Synonymous	1 (0.28)
Stop loss	1 (0.28)
Total	362

Supplementary Table 3– List of samples having biallelic *GJB2* mutations in the Southern Chinese population

The table shows the list of samples confirmed with biallelic *GJB2* mutations. Phasing was detected in trans because it could be determined through short-read sequencing reads. Nineteen samples were shown to be GJB2 c.109G>A homozygous mutations, 2 samples were compound heterozygous due to c.109G>A and c.235delC, and 1 sample was compound heterozygous due to c.109G>A and c.230G>A.

**Number of sample(s)**	***GJB2* mutations**	**Protein changes**
1	c.[109G>A];[230G>A]	p.[(Val37Ile)];[(Trp77Ter)]
2	c.[109G>A];[235delC]	p.[(Val37Ile)];[(Leu79CysfsTer3)]
19	c.[109G>A];[109G>A]	p.[(Val37Ile)];[(Val37Ile)]
